# Dietary Complex Probiotic Supplementation Changed the Composition of Intestinal Short-Chain Fatty Acids and Improved the Average Daily Gain of Growing Pigs

**DOI:** 10.3390/vetsci10020079

**Published:** 2023-01-21

**Authors:** Juan Wang, Shuwei Li, Wenjie Tang, Hui Diao, Hongfu Zhang, Honglin Yan, Jingbo Liu

**Affiliations:** 1 School of Life Science and Engineering, Southwest University of Science and Technology, Mianyang 621010, China; 2 Sichuan Academy of Animal Science, Chengdu 610066, China; 3 State Key Laboratory of Animal Nutrition, Institute of Animal Sciences, Chinese Academy of Agricultural Sciences, Beijing 100193, China

**Keywords:** growing pigs, probiotics, short-chain fatty acids, growth performance

## Abstract

**Simple Summary:**

In this research, we studied the effects of complex probiotic (*Bacillus licheniformis*, *Bacillus subtilis*, *Lactobacillus acidophilus* and *Saccharomyces cerevisiae*) on growth performance, nutrient digestibility, blood characteristics, fecal microbiota, fecal short-chain fatty acids, fecal score and fecal gas emissions in growing pigs. Dietary complex probiotic supplementation changed the composition of intestinal short-chain fatty acids, improved growth performance and reduced harmful gas emissions in growing pigs. This study provides basic data for the use of compound probiotic.

**Abstract:**

At present, probiotics are being extensively evaluated for their efficacy as an alternative to antibiotics, and their safety in livestock production. In this study, 128 (Duroc, Yorkshire and Landrace) pigs with an average initial body weight of 28.38 ± 0.25 kg were allocated to four dietary treatments in a randomized complete-block design. There were eight pens per treatment, with four pigs per pen (two barrows and two gilts). Dietary treatments included: (1) control diet; (2) control diet + 0.05% complex probiotic; (3) control diet + 0.1% complex probiotic; (4) control diet + 0.2% complex probiotic. During the 28-day experimental period, the feeding of 0.1% complex probiotic in the diet increased body weight and average daily gain (*p* < 0.05). The addition of complex probiotics decreased total cholesterol and glucose concentrations in the blood (*p* < 0.01). Acetate concentrations in the blood increased from 0.1% complex probiotic in the diet (*p* < 0.05), while NH_3_ and H_2_S emissions in the feces decreased (*p* < 0.05) from 0.1% or 0.2% complex probiotic in the diet. In conclusion, dietary complex probiotic supplementation changed the composition of intestinal short-chain fatty acids and improved growth performance for growing pigs.

## 1. Introduction

As an active microbial complex, probiotics can effectively enhance the positive effects of gut microbes on the host, regulate gut health and improve production performance [1,2]. The microorganisms in probiotics can produce vitamins, amino acids, organic acids, enzymes, and other substances, through their own metabolism. These substances can boost nutrient absorption, improve gut health and have antibacterial properties. In recent years, probiotics have attracted extensive attention as a dietary alternative to feeding antibiotics and nutritional additives [1,2,3,4,5,6]. In addition, adding probiotics can reduce the emissions of harmful fecal gases and the pollution problem from intensive farming [7].

The type and dose of probiotics, the mechanism of action for microbial strains, and the interactions with the host are important to determine the effects of probiotics [8,9]. The strains currently used as probiotic additives mainly include *Bacillus licheniformis*, *Bacillus subtilis*, *Lactobacillus acidophilus* and *Saccharomyces cerevisiae*. Some selected specific strains are also used for the preparation of probiotics [10,11], such as *Lactobacillus casei*, *Lactobacillus plantarum* and *Lactobacillus johnsonii BS15*. There have been several studies adding one microbial species or a combination of several microbial species as a probiotic [12,13,14]. However, there are few studies examining the effects of the previously mentioned microbial species (*Bacillus licheniformis*, *Bacillus subtilis*, *Lactobacillus acidophilus*, and *Saccharomyces cerevisiae*.) for probiotic supplementation in growing pigs. Therefore, the objective of this study was to evaluate the effects of different concentrations of complex probiotic (contains four mixed probiotics) on growth performance, nutrient digestibility, blood profile, fecal microbiota, fecal short-chain fatty acids (SCFAs), fecal score and fecal gas emissions in growing pigs.

## 2. Materials and Methods

The animal study protocol was approved by the Institutional Review Board of the Southwest University of Science and Technology (protocol code SM00155). The complex probiotic product we have used comprises four kinds of mixed probiotics. The effective content of each probiotic was 5.1 × 10^7^ CFU/g *Bacillus licheniformis*, 6.3 × 10^7^ CFU/g *Bacillus subtilis*, 4.3 × 10^7^ CFU/g *Lactobacillus acidophilus* and 2.5 × 10^7^ CFU/g *Saccharomyces cerevisiae*.

### 2.1. Experimental Design, Animals and Housing

One hundred and twenty-eight (Duroc, Landrace and Yorkshire) growing pigs (average initial body weight (BW) of 28.38 ± 0.25 kg) were used in a 28-day growth trial. At the start of the experiment, pigs were assigned to four dietary treatments based on initial BW in a randomized complete-block design. There were eight replicate pens per dietary treatment, with four pigs per pen (2 barrows, 2 gilts). Dietary treatments included: (1) CON (control diet) with no probiotic, 0%; (2) CON with 0.05% complex probiotic; (3) CON with 0.1% complex probiotic; (4) CON with 0.2% complex probiotic. The control diet (Table 1) was a compound feed in mash, formulated to meet nutrient requirements for 25–50 kg growing pigs [15]. Throughout the experiment, the pigs were housed in plastic floor pens and all the pigs were provided with ad libitum access to feed and water through a self-feeder and a nipple drinker, respectively. The target room temperature and humidity throughout the study were 20 °C and 60%, respectively.

### 2.2. Fecal Score and Sample Collection

Feces were scored on a scale from one to five (fecal score: 1 hard, dry pellet; 2 firm, formed stool; 3 soft, moist stool that retains shape; 4 soft, unformed stool that assumes the shape of the container; 5 watery liquid that can be poured) by observing the morphology of the feces in each pen at 08:00 and 20:00 every day. BW for each pig was measured on days 0, 14 and 28, to determine average daily gain (ADG). Feed consumption was recorded every day, by measuring the amount of feed fed and remaining on a pen, to calculate average daily feed intake (ADFI), and feed to gain ratio (F: G). Chromium oxide was added to the diet as an indigestible marker at 0.5% for 4 days (during days 11 to 14 and days 25 to 28) prior to fecal collection on days 14 and 28 for calculating dry matter (DM), crude protein (CP) and gross energy (GE) digestibility values. On day 14, rectal massage was used on each pig to collect 300 g of fresh feces for the determination of fecal gas emissions, with another 60 g of fresh feces used to determine nutrient digestibility. Blood samples were taken from eight pigs per dietary treatment. One pig was randomly selected from each pen (guaranteed male to female ratio of 1:1 for each treatment) and bled via jugular venipuncture at the end of the experiment. On day 28, a large amount of feces was collected through rectal massaging. Collection of 300 g and 60 g of fresh feces in the front section (first discharge the feces within 15 cm) was carried out to measure fecal gas emissions and nutrient digestibility, respectively. After collection, 20 g of fresh feces in the latter section (more than 15 cm in length) were stored in a cryopreservation tube at −80 °C, to measure the microbial content of the feces, and 40 g of those were placed in a sample tube at −20 °C to measure the SCFAs in the feces. After fecal collection from each pig was completed, feces from all the pigs in the same pen were mixed prior to analysis.

### 2.3. Nutrient Digestibility and Blood Profile Analyses

Diet and fecal samples were analyzed after drying in a forced-air oven and grinding through a 1 mm sieve. Calcium (Ca) and phosphorus (P) were determined in the diet based on the method of Liu et al. [16]. Feces were dried in a forced-air oven at 105 °C for 2 h [17] to determine the DM content. The concentrations of CP in experimental diets and feces were determined by the combustion method [17]. Dietary and fecal GE were determined by a fully automated calorimeter (BYLRY-3000W). After nitrification, Cr_2_O_3_ concentrations in diets and fecal samples were determined by reading absorbance at 450 nm on a spectrophotometer. Blood indicators were measured by using an automatic biochemical analyzer (BK-600, biobased, Jinan City, Shandong Province, China) [17].

### 2.4. Fecal Microbiota Analysis

Fecal microbial assays were performed as described by Liu et al. [18]. Fecal total DNA was extracted by using Omega’s stool DNA isolation kit (Omega BioTec, Norcross, GA, USA). In this experiment, the concentrations of six species of bacteria, including *Prevotella*, *Bacteroides*, *Bifidobacterium*, *Escherichia coli*, *Lactobacillus* and *Bacillus*, were determined. Primer sequences and annealing temperatures were shown in Table 2. This test used 11 μL in a fluorescence-quantitative PCR reaction system, including 2 μL of DNA, 2.7 μL of RNase-free water, 0.4 μL (10 μM) of upstream and downstream primers and 5.5 μL 2× KAPA SYBR FAST qPCR Kit Master Mix. The reaction conditions were: 50 °C for 2 min, 95 °C for 10 min, 40 cycles of denaturation/annealing (95 °C for 15 s, 60 °C for 1 min) and a melting curve process (from 70 °C to 90 °C, increasing by 0.5 °C every 5 s). The fluorescence-quantitative reaction was performed on an ABI 7600 instrument.

### 2.5. Determination of SCFAs in Feces

Fecal samples were sealed, thawed at 5 °C for 4 h and then mixed. After weighing 15 g of feces, 15 mL of distilled water was added for mixing, then the mixture was centrifuged at 4000× *g* and 18 °C for 5 min. Five mL of supernatant was then added to 5 mL of HCl, the mixture then being stored at 7 °C. This mixture was centrifuged at 14,000× *g* and 17 °C for 10 min then injected into a gas chromatograph for detection. The column (1.8 m) with the stationary phase SP 1200 (Supelco) contained 10% SP 1200, 1% H_3_PO_4_, and acid-washed 80/100 Chromosorb W. Flame ionization detector was used for detection, with nitrogen used as a carrier gas.

### 2.6. Fecal Gas Emissions Analyses

The fresh feces (300 g) from each pen were placed in separate 2.6 L plastic boxes with a small hole located in the middle of one side, which was sealed with adhesive plaster. The samples were fermented for 24 h at room temperature (25 °C). Afterwards, 100 mL of the head-space air was sampled from approximately 2.0 cm above the fecal sample. After the gas was collected, the small hole was resealed for gas detection. Before measurement, the fecal samples were manually shaken for approximately 30 s to disrupt crust formation on the surface of the fecal samples and to homogenize the samples. Concentrations of NH_3_, H_2_S, mercaptan, acetic acid and CO_2_ were measured by using a portable 6-in-1 gas detector (SGA-606, ingoan, Shenzhen, Guangdong Province, China).

### 2.7. Statistical Analyses

Data was analyzed by ANOVA, using the GLM procedure of SAS (SAS Inst. Inc., Cary, NC, USA) for a randomized complete-block design evaluating the level of complex probiotic added to the diet. The dose–response effects of dietary complex probiotic were computed using orthogonal polynomial contrasts for evaluating linear and quadratic effects. Post-hoc test was used to compare the control group (0 % complex probiotic) versus complex probiotic added to the diet. For all response criteria, the pen served as the experimental unit. Variability in the data was expressed as the pooled SE and *p* < 0.05 was considered statistically significant.

## 3. Results

### 3.1. Growth Performance and Nutrient Digestibility

There were linear increases in final body weight (FBW; Day 28) and ADG (Day 15–28 and Day 0–28) as the amounts of dietary complex probiotic increased (Table 3; *p* < 0.01), with trait values greater for pigs fed 0.1% complex probiotic than CON pigs (*p* < 0.05). Linear increases in average daily feed intakes (ADFI) (Day 15–28 and Day 0–28) were present as the amounts of dietary complex probiotic increased (Table 3; *p* < 0.04), but did not affect (*p* > 0.12) ADFI or F: G values versus CON pigs. There was no effect of dietary complex probiotic supplementation on nutrient digestibility in growing pigs (*p* > 0.05) when assessed on day 14 or day 28 (Table 4).

### 3.2. Blood Profile

The addition of complex probiotic to the diet reduced (*p* < 0.01) total cholesterol and glucose concentrations in the blood (Table 5). There was a linear decrease (*p* < 0.02) in total cholesterol and glucose concentrations as the amounts of dietary complex probiotic increased, with total cholesterol and glucose concentrations further decreasing (*p* < 0.05) when complex probiotic increased from 0.05% to 0.1% of the diet. Dietary complex probiotic supplementation did not affect (*p* > 0.38) the concentrations of HDL, LDL, triacylglycerides, total protein and urea nitrogen in the blood.

### 3.3. Fecal Microbiota, Fecal SCFAs, Fecal Score and Fecal Gas Emissions

There was no effect (*p* > 0.07) of dietary complex probiotic supplementation on the fecal score in growing pigs throughout the study. (Table 6). Concentrations of *Bifidobacterium* increased (*p* < 0.04) with the addition of dietary complex probiotic, along with a linear increase (*p* = 0.01) in *Bifidobacterium* numbers as the amount of dietary complex probiotic increased (Table 7). There was no effect (*p* > 0.27) of dietary complex probiotic supplementation on the concentrations of any other microbial species evaluated (Table 7). Acetate was the only short-chain fatty acid to increase (*p* < 0.02) when complex probiotic were added to the diet (Table 8). There were linear increases (*p* < 0.05) in acetate, butyrate and total SCFA concentrations as the amounts of complex probiotic increased in the diet (Table 8). The addition of complex probiotic to the diet decreased (*p* < 0.04) concentrations of NH_3_ on day 14, and concentrations of NH_3_ and H_2_S on day 28 (Table 9), with linear decreases (*p* < 0.03) for NH_3_ and H_2_S as the amount of dietary complex probiotic increased. Ammonia concentrations on day 28 further decreased when the dietary inclusion rate for complex probiotic increased from 0.05% to 0.1% (Table 9).

## 4. Discussion

The results of this study showed that although dietary supplementation of complex probiotic had no significant effect on nutrient digestibility and feces score of growing pigs, an appropriate dose of complex probiotic (0.1%) could improve the ADG during days 15 to 28, change the composition of intestinal microorganisms and SCFAs, and reduce the incidence of noxious gas emissions in growing pigs.

Growth performance is an intuitive comprehensive assessment of animal production, affected by the ambient environment and diet composition. The nutritional composition of the diet [25,26] including dietary Ca and P concentrations [16,27,28], along with feeding management [29,30,31], are important factors for pig growth performance. There have been many studies evaluating the effects of probiotics on the growth performance of pigs. Meng et al. [4] indicated that dietary supplementation of probiotics (*Bacillus subtilis*-endospore and *Clostridium butyricum*-endospore complex) could effectively improve the ADG and lower F: G. Another study showed that 0.3% and 0.5% EM^®®^ probiotics (*Saccharomyces cerevisiae*, *Lactobacillus casei*, *Lactobacillus plantarum*) supplementation had lower ADG compared to the 0% probiotic group in the last four weeks before slaughter [10]. Using complex probiotic (*Lactobacillus plantarum*, *Lactobacillus fermentum* and *Enterococcus faecium*) improved the daily weight gain of weaned piglets [32]. The results from the present found adding 0.1% complex probiotic to the diet could significantly increase ADG with no effects on ADFI and F: G, versus pigs fed a control diet with no probiotics added. The addition for probiotics effectively increased FBW of growing pigs compared to the control group. After comparing the results of each of the above experiments, it can be seen that the effects of adding compound probiotics on growth performance were both improved and reduced. The reason for the different results (described above) from trial to trial could be the difference in the type and dose of probiotics added.

Although we observed an increase in ADG in the present study, feeding probiotics did not affect nutrient digestibility [33,34,35,36]. To uncover clues about the effects of probiotics on growth performance, we analyzed the hindgut microbes, as well as SCFAs. Our results showed that feeding probiotics in the diet increased numbers of *Bifidobacteria,* while did not affect the numbers of several microbial species. Preliminary study showed *Bifidobacteria* could reduce diarrhea rates [37]. Fecal scores for this study showed no significant difference among the treatment groups, because no diarrhea results were observed. Changes in the composition of gut microbes often affect the composition of their products [38]. We found that adding 0.1% probiotics to the diet could significantly increase amounts of acetate and butyrate produced in the feces. Butyrate acid and acetate could be absorbed by the hindgut and provide a large amount of energy for the body [39]. This is probably the main reason for the improvement in ADG by adding complex probiotic. In the present study, adding probiotics to the diet significantly reduced concentrations of total cholesterol and glucose. Previous studies have found that higher levels of SCFAs could significantly reduce plasma glucose and cholesterol levels [40,41]. SCFAs could regulate plasma glucose and cholesterol levels through the hepatic AMPK pathway, and also affect plasma glucose levels by increasing gut satiety hormones. In this study, we also found that the content of total SCFAs in the probiotic group tended to increase, but not significantly. The above results indicated that the addition of probiotics could change the composition of intestinal microorganisms and SCFAs, thereby affecting the levels of plasma glucose and cholesterol, altered pathways of nutrient metabolism, and the final manifestation was the improvement of ADG and FBW. More evidences of the effects on nutrient metabolism need further collection.

With advancements in intensive farming, pollution from the gaseous emissions from pig manure is a serious problem. This study found that adding probiotics could effectively reduce NH_3_ and H_2_S emissions from manure. High concentrations of NH_3_ and H_2_S damaged the respiratory mucosa of pigs and reduced growth performance [42]. Adding probiotics could effectively improve the air environment on pig farms and had a positive effect on the control of farm pollution.

## 5. Conclusions

Dietary supplementation of 0.5% complex probiotic had limited effects on growth performance in growing pigs. Dietary supplementation of 0.1% and 0.2% complex probiotic altered gut microbial composition and SCFAs content, improved ADG and reduced noxious gas emissions.

## Figures and Tables

**Table 1:** Ingredient and chemical composition of diets.

Ingredients, g/kg	
Corn	700.0
Soybean meal (43% CP)	225.0
Wheat bran	30.0
Soybean oil	15.0
Monocalcium phosphate	12.0
Limestone (38% Ca)	7.0
Salt	3.0
Choline chloride (50%)	1.0
L-Lys, 78.8%	2.8
DL-Met, 98%	0.5
L-Thr, 97.5%	1.2
Vitamin premix ^1^	1.5
Mineral premix ^2^	1.0
Total	1000
Determined nutrients, %	
DE, kcal/kg	3390
Crude protein	15.31
Ca	0.656
Total P	0.55
STDD P	0.38
NDF	9.43
Ca: P	1.19

^1^ Vitamin premix contained per gram of premix: vitamin A, 2640 IU; vitamin D_3_, 264 IU; vitamin E, 17.6 IU; vitamin K activity, 2.4 mg; menadione, 880 μg; vitamin B_12_, 15.4 μg; riboflavin, 3.52 mg; D-pantothenic acid, 8.8 mg; niacin, 13.2 mg. ^2^ Mineral premixes contained per gram of premix: Cu (as copper chloride), 9 mg; I (as ethylenediamine dihydroiodide (EDDI)), 0.36 mg; Fe (as ferrous carbonate), 194 mg; Mn (as manganese oxide), 17 mg; and Zn (as zinc oxide), 149 mg.

**Table 2:** Primer sequence for fecal microbiota.

Bacteria	Upstream Primer (5′ → 3′)	Downstream Primers (5′ → 3′)	AT, °C	R
*Prevotella*	CACCAAGGCGACGATCA	GGATAACGCCTGGACCT	60	[19]
*Bacteroides*	CGATGGATAGGGGTTCTGAGAGGA	GCTGGCACGGAGTTAGCCGA	60	[20]
*Bifidobacterium*	TCGCGTCYGGTGTGAAAG	GGTGTTCTTCCCGATATCTACA	60	[21]
*Escherichia coli*	CATGCCGCGTGTATGAAGAA	CGGGTAACGTCAATGAGCAAA	60	[22]
*Lactobacillus*	AGCAGTAGGGAATCTTCCA	ATTCCACCGCTACACATG	60	[23]
*Bacillus*	CCTACGGGAGGCAGCAGTAG	GCGTTGCTCCGTCAGACTTT	59	[24]

Note. AT, annealing temperature; R, reference.

**Table 3:** Effects of dietary complex probiotic supplementation on growth performance in growing pigs.

Item	Probiotics (% of Diet)				*p*-Value		
	0 (CON)	0.05	0.1	0.2	CON vs. Probiotics	Linear	Quadratic
Body weight, kg							
Initial	28.26 ± 0.26	28.43 ± 0.28	28.38 ± 0.23	48.46 ± 0.15	0.575	0.753	0.731
Day 14	38.00 ± 0.16	38.18 ± 0.36	38.24 ± 0.30	38.09 ± 0.29	0.628	0.585	0.881
Day 28	48.38 ± 0.31 ^b^	48.71 ± 0.32 ^ab^	49.85 ± 0.31 ^a^	48.99 ± 0.37 ^ab^	0.036	0.005	0.359
ADG, kg/day							
Day 0–14	695 ± 21.7	696 ± 34.9	704 ± 21.4	687 ± 18.6	0.984	0.815	0.914
Day 15–28	741 ± 13.4 ^b^	752 ± 31.3 ^b^	829 ± 11.2 ^a^	778 ± 7.78 ^ab^	0.074	0.004	0.185
Day 0–28	718 ± 11.7 ^b^	724 ± 8.69 ^ab^	766 ± 12.3 ^a^	733 ± 12.4 ^ab^	0.135	0.009	0.235
ADFI, kg/day							
Day 0–14	1515 ± 42.7	1502 ± 78.9	1490 ± 36.6	1861 ± 22.2	0.613	0.746	0.997
Day 15–28	1793 ± 40.6	1815 ± 79.2	1977 ± 29.3	1861 ± 22.3	0.153	0.017	0.279
Day 0–28	1654 ± 29.2	1659 ± 21.2	1733 ± 17.8	1660 ± 24.0	0.340	0.034	0.265
F: G							
Day 0–14	2.18 ± 0.02	2.16 ± 0.01	2.12 ± 0.03	2.12 ± 0.02	0.058	0.049	0.814
Day 15–28	2.42 ± 0.02	2.41 ± 0.03	2.38 ± 0.02	2.39 ± 0.01	0.378	0.293	0.726
Day 0–28	2.30 ± 0.01	2.29 ± 0.02	2.26 ± 0.02	2.27 ± 0.01	0.121	0.092	0.711

Note. ADFI, average daily feed intake; ADG, average daily gain; F:G, the ratio of feed to gain; a, b Means in the same row with different superscripts differ significantly (*p* < 0.05).

**Table 4:** Effects of dietary complex probiotic supplementation on nutrient digestibility in growing pigs.

Item, %	Probiotics (% of Diet)				*p*-Value		
	0 (CON)	0.05	0.1	0.2	CON vs. Probiotics	Linear	Quadratic
Day 14							
Dry matter	84.99 ± 0.60	84.48 ± 0.72	84.78 ± 0.77	83.89 ± 0.85	0.502	0.851	0.679
Crude protein	83.69 ± 0.79	83.51 ± 0.94	82.89 ± 1.24	82.59 ± 0.93	0.566	0.598	0.864
Gross energy	83.65 ± 0.94	83.85 ± 0.74	82.99 ± 0.95	82.85 ± 0.83	0.691	0.618	0.644
Day 28							
Dry matter	82.99 ± 0.84	83.22 ± 0.93	82.63 ± 0.91	82.53 ± 1.02	0.860	0.798	0.736
Crude protein	81.59 ± 0.96	80.67 ± 0.81	81.50 ± 0.92	81.88 ± 1.04	0.831	0.952	0.457
Gross energy	83.41 ± 0.73	84.35 ± 0.69	83.09 ± 0.61	82.96 ± 0.71	0.949	0.756	0.230

**Table 5:** Effects of dietary complex probiotic supplementation on the blood profile in growing pigs.

Item	Probiotics (% of Diet)				*p*-Value		
	0 (CON)	0.05	0.1	0.2	CON vs. Probiotics	Linear	Quadratic
Total cholesterol, mmol/L	2.41 ± 0.03 ^a^	2.36 ± 0.02 ^a^	2.25 ± 0.03 ^b^	2.24 ± 0.03 ^b^	0.001	0.0003	0.385
HDL, mmol/L	0.99 ± 0.03	0.96 ± 0.04	1.01 ± 0.03	1.03 ± 0.04	0.839	0.707	0.387
LDL, mmol/L	1.05 ± 0.06	1.09 ± 0.05	1.08 ± 0.03	1.08 ± 0.05	0.607	0.718	0.729
Triacylglycerides, mmol/L	0.47 ± 0.03	0.45 ±0.04	0.44 ±0.04	0.46 ±0.04	0.632	0.614	0.856
Glucose, mmol/L	7.07 ± 0.30 ^a^	6.13 ± 0.30 ^b^	5.76 ± 0.33 ^c^	5.86 ± 0.35 ^c^	0.006	0.011	0.505
Total protein, g/L	70.05 ± 1.06	70.83 ± 1.28	69.84 ± 1.07	71.03 ± 1.08	0.709	0.902	0.555
Urea nitrogen, mmol/L	4.01 ± 0.19	3.91 ± 0.28	3.96 ± 0.27	4.00 ± 0.39	0.876	0.913	0.835

Note. HDL, high-density lipoprotein; LDL, low-density lipoprotein; a, b, c Means in the same row with different superscripts differ significantly (*p* < 0.05).

**Table 6:** Effects of dietary complex probiotic supplementation on fecal score in growing pigs.

Item	Probiotics (% of Diet)				*p*-Value		
	0 (CON)	0.05	0.1	0.2	CON vs. Probiotics	Linear	Quadratic
Day 7	3.37 ± 0.06	3.23 ± 0.12	3.43 ± 0.06	3.49 ± 0.02	0.931	0.638	0.089
Day 14	3.42 ± 0.05	3.35 ± 0.06	3.43 ± 0.06	3.40 ± 0.06	0.760	0.865	0.332
Day 21	3.34 ± 0.06	3.52 ± 0.04	3.42 ± 0.06	3.44 ± 0.0.7	0.099	0.354	0.073
Day 28	3.39 ± 0.04	3.45 ± 0.06	3.28 ± 0.03	3.09 ± 0.12	0.247	0.323	0.258

Note. Fecal score = 1 hard, dry pellet; 2 firm, formed stool; 3 soft, moist stool that retains shape; 4 soft, unformed stool that assumes the shape of the container; 5 watery liquid that can be poured.

**Table 7:** Effects of dietary complex probiotic supplementation on fecal microbiota in growing pigs.

Item, Lg (Copies/g)	Probiotics (% of Diet)				*p*-Value		
	0 (CON)	0.05	0.1	0.2	CON vs. Probiotics	Linear	Quadratic
*Prevotella*	10.59 ± 0.24	10.34 ± 0.18	10.37 ± 0.21	10.40 ± 0.27	0.418	0.523	0.651
*Bacteroides*	11.79 ± 0.21	11.76 ± 0.20	11.63 ± 0.31	11.78 ± 0.21	0.824	0.663	0.863
*Bifidobacterium*	11.80 ± 0.36	12.01 ± 0.29	13.19 ± 0.33	13.10 ± 0.37	0.036	0.010	0.273
*Escherichia coli*	10.64 ± 0.24	10.78 ± 0.31	10.95 ± 0.28	10.84 ± 0.44	0.581	0.536	0.979
*Lactobacillus*	7.60 ± 0.17	7.65 ± 0.18	7.55 ± 0.19	7.51 ± 0.14	0.896	0.864	0.733
*Bacillus*	8.48 ± 0.26	8.35 ± 0.19	8.32 ± 0.18	8.52 ± 0.17	0.719	0.583	0.860

**Table 8:** Effects of dietary complex probiotic supplementation on fecal SCFAs in growing pigs.

Item, mmol/L	Probiotics (% of Diet)				*p*-Value		
	0 (CON)	0.05	0.1	0.2	CON vs. Probiotics	Linear	Quadratic
Lactate	2.48 ± 0.14	2.60 ± 0.16	2.66 ± 0.20	2.70 ± 0.25	0.466	0.558	0.893
Acetate	60.29 ± 3.66 ^b^	65.76 ± 2.44 ^ab^	72.98 ± 2.79 ^a^	71.36 ± 2.66 ^ab^	0.013	0.008	0.821
Propionate	18.98 ± 0.77	18.51 ± 1.80	18.21 ± 1.24	17.94 ± 1.27	0.573	0.652	0.956
Isobutyrate	2.74 ± 1.77	2.86 ± 0.23	2.91 ± 0.19	2.90 ± 0.23	0.537	0.580	0.891
Butyrate	8.14 ± 0.70	8.23 ± 0.81	10.90 ± 0.90	10.91 ± 0.94	0.100	0.038	0.251
Isovalerate	5.29 ± 0.45	5.48 ± 0.48	5.58 ± 0.52	5.71 ± 0.42	0.595	0.687	0.944
Valerate	3.64 ± 0.27	3.68 ± 0.24	3.93 ± 0.34	3.95 ± 0.27	0.536	0.506	0.776
Total SCFAs	99.07 ± 4.61	104.5 ± 4.17	114.5 ± 5.60	112.8 ± 4.82	0.063	0.044	0.722

Note. SCFAs, short chain fatty acids; a, b Means in the same row with different superscripts differ significantly (*p* < 0.05).

**Table 9:** Effects of dietary complex probiotic supplementation on fecal gas emissions in growing pigs.

Item, ppm	Probiotics (% of Diet)				*p*-Value		
	0 (CON)	0.05	0.1	0.2	CON vs. Probiotics	Linear	Quadratic
Day 14							
Methyl mercaptans	4.72 ± 0.11	4.74 ± 0.17	4.84 ± 0.18	4.82 ± 0.19	0.697	0.634	0.837
NH_3_	4.00 ± 0.13 ^a^	3.62 ± 0.14 ^ab^	3.38 ± 0.17 ^b^	3.43 ± 0.15 ^ab^	0.007	0.010	0.703
H_2_S	2.75 ± 0.11 ^a^	2.71 ± 0.10 ^a^	2.42 ± 0.14 ^ab^	2.20 ± 0.14 ^b^	0.074	0.086	0.432
Acetic acid	6.88 ± 0.19	6.79 ± 0.23	6.68 ± 0.20	6.74 ± 0.26	0.589	0.562	0.988
CO_2_	10,813 ± 226	10,953 ± 211	10,848 ± 355	10,969 ± 326	0.751	0.935	0.751
Day 28							
Methyl mercaptans	4.97 ± 0.23	5.13 ± 0.19	5.00 ± 0.25	5.05 ± 0.26	0.750	0.933	0.640
NH_3_	4.16 ± 0.14 ^a^	4.05 ± 0.11 ^a^	3.47 ± 0.14 ^b^	3.36 ± 0.14 ^b^	0.010	0.002	0.184
H_2_S	3.06 ± 0.14 ^a^	2.99 ± 0.13 ^ab^	2.51 ± 0.11 ^b^	2.50 ± 0.12 ^b^	0.031	0.025	0.269
Acetic acid	7.37 ± 0.26	7.48 ± 0.25	7.39 ± 0.22	7.33 ± 0.29	0.932	0.972	0.768
CO_2_	12,591 ± 243	12,700 ± 258	12,716 ± 330	12,575 ± 291	0.832	0.772	0.902

Note. a, b Means in the same row with different superscripts differ significantly (*p* < 0.05).

## Data Availability

Data will be made available upon reasonable request.
